# Identification of differential expressed PE exosomal miRNA in lung adenocarcinoma, tuberculosis, and other benign lesions

**DOI:** 10.1097/MD.0000000000008361

**Published:** 2017-11-03

**Authors:** Yan Wang, Yan-Mei Xu, Ye-Qing Zou, Jin Lin, Bo Huang, Jing Liu, Jing Li, Jing Zhang, Wei-Ming Yang, Qing-Hua Min, Shu-Qi Li, Qiu-Fang Gao, Fan Sun, Qing-Gen Chen, Lei Zhang, Yu-Huan Jiang, Li-Bin Deng, Xiao-Zhong Wang

**Affiliations:** a Department of Clinical Laboratory, The Second Affiliated Hospital of Nanchang University, Jiangxi; b Department of Hematology, The Affiliated Hospital of Guizhou Medical University, Guizhou; c The Key Laboratory of Molecular Medicine, The Second Affiliated Hospital of Nanchang University; d Department of Clinical Laboratory, The First Affiliated Hospital of Nanchang University; e Institute of Translational Medicine, Nanchang University, Jiangxi, China.

**Keywords:** deep sequencing, exosome, miRNA, pleural effusions

## Abstract

Pleural effusion (PE) is a common clinical complication of many pulmonary and systemic diseases, including lung cancer and *tuberculosis*. Nevertheless, there is no clinical effective biomarker to identify the cause of PE. We attempted to investigate differential expressed exosomal miRNAs in PEs of lung adenocarcinoma (APE), *tuberculous* (TPE), and other benign lesions (NPE) by using deep sequencing and quantitative polymerase chain reaction (qRT-PCR). As a result, 171 differentiated miRNAs were observed in 3 groups of PEs, and 11 significantly differentiated exosomal miRNAs were validated by qRT-PCR. We identified 9 miRNAs, including miR-205-5p, miR-483-5p, miR-375, miR-200c-3p, miR-429, miR-200b-3p, miR-200a-3p, miR-203a-3p, and miR-141-3p which were preferentially represented in exosomes derived from APE when compared with TPE or NPE, while 3 miRNAs, including miR-148a-3p, miR-451a, and miR-150-5p, were differentially expressed between TPE and NPE. These different miRNAs profiles may hold promise as biomarkers for differential diagnosis of PEs with more validation based on larger cohorts.

## Abstract 2

Supplemental Digital Content is available in the text

## 1 Introduction

Pleural effusion (PE) is common but abnormal accumulation of fluid in the pleural space. Lung cancer and *tuberculosis* are the 2 most frequent causes of exudative PEs, suggesting pleural involvement of lesions.^[1,2]^ Nonsmall cell lung cancer (NSCLC) accounts for more than 80% of lung cancer types and these include adenocarcinoma (AC) and squamous cell carcinoma (SCC).^[3]^ Lack of specific clinical presentations and biochemical markers complicate the process of distinguishing of PE etiology.

Using Light's criteria to classify PEs into exudates and transudates is a preliminary step and then other diagnostic methods, such as conventional diagnostic methods, can be used characterize PEs.^[2,4–6]^ Cytology can be used to confirm 30% to 60% malignant PEs in advanced disease stages but constant variation depends on tumor origin confused us. Immunocytochemistry can be used to study pleura, but sensitivity or specificity is not optimal.^[7]^ Histological diagnosis and better imaging can help when combined with thoracoscopy, but this aggressive and invasive approach can cause complications and increase morbidity as well. Soluble mediators were also found to contribute to PEs: vascular endothelial growth factor (VEGF), tumor necrosis factor (TNF), endothelin,^[8]^ lnterleukin (IL)-27,^[9]^ and IL-6.^[10–12]^ These markers are more sensitive and specific than conventional diagnostic methods, while its accuracy is limited when PE is of diverse sources and markers may be difficult to measure. In addition, the markers are poorly stable as disease indicators, which may cause misdiagnosis due to degradation. Thus, ≈30% of PE is attributed to unknown causes. Recently, circulating exosomes for differential diagnosis has drawn considerable attention.

Exosomes, membrane-bound vesicles of 30 to 100 nm diameter, occur in almost all biological fluids.^[13]^ They are homogeneous in size and can be differentiated from extracellular vesicles (EVs) such as microvesicles and large oncosomes.^[14,15]^ Exosomes can mediate the exchange of intricate intercellular messages with highly heterogeneous signaling factors, including miRNAs, mRNAs, DNA, and proteins.^[16]^ Numerous studies have revealed exosomal donor-cell-specific signatures,^[17–19]^ and its nature and abundance of the molecular cargo are often influenced by donor cell pathological status and origin.^[20]^ Besides, exosomal miRNAs can exist stably in body fluids, and related to information of maternal tissue or cell based on miRNA expression and composition.^[21,22]^ Intriguingly, exosomal miRNAs likely interact with RNA-induced silencing complexes (RISC), mediating miRNA formation and sorting.^[23,24]^ AGO2 knockout was reported to change the types or abundance of preferentially exported miRNA,^[25]^ so circulating exosomal miRNAs may be potential noninvasive diagnostic biomarkers.^[26–28]^

We previously deep sequenced EVs derived from PEs of NSCLC and tuberculosis, acquired a group of differential expressed miRNAs.^[29]^ However, the results were conflicting due to EVs heterogeneity and generalized histological subtypes.^[30,31]^ Relative to EVs, exosomes are better as the liquid biopsy specimens for their homogeneity in size and contents (Extended Table 1). To identify the potential biomarkers to differentiate NSCLC, AC, and SCC we collected 3 groups of exosomes rather than EVs for deep sequencing and qRT-PCR analysis. Our study would contribute to differentiate diagnosis of these diseases.

## 2 Materials and methods

### 2.1 Patients

The study protocol was approved by the Ethics Committee of the Second Affiliated Hospital of Nanchang University, China, and informed consent was obtained from each patient. PE samples were collected before clinical treatment from November 2013 to November 2015 at the Second Affiliated Hospital of Nanchang University. The eligible PE samples were selected according to the following included criteria: exudate based on Light's criteria;^[2,32]^ adenocarcinoma tumor cells in PEs or in pleural biopsy specimens were defined as APE; the presence of acid fast bacilli and *M tuberculosis* in PEs met criteria for TPE; undiagnosed exudates negative for cancer according to histological or cytological criteria were classified as benign controls (NPE). Meanwhile, PE samples were excluded when patients were diagnosed as atelectasis, pulmonary embolism, obstructive pneumonia, or other primary tumor diseases. For each patient, an initial diagnostic thoracenteses was performed when patients sitting upright after local infiltration with lignocaine. PE was obtained via needle aspiration and aspirate volumes were recorded.

### 2.2 Exosome precipitation

Exosomes were isolated from 120 mL PEs with differential centrifugations as previously described.^[29]^ To remove cells and debris, PE supernatants were sequentially centrifuged for 20 minutes at 1200 × g and 30 minutes at 10,000 × g. To remove particles greater than 200 nm, supernatants were filtered through 0.22 μm pore filters and rinsed with PBS using ultracentrifugation (Optima L-80XP Ultracentrifuge, Beckman Coulter, Beckman) for 1 hours at 120,000 × g twice. All the steps were carried out at 4°C. Finally, the pellets were resuspended in PBS and stored at −80°C for further use.

### 2.3 Scanning electron microscopy (SEM) and nanoparticle analysis (NPA)

SEM (FEI XL30, The Netherlands) was used to visualize exosome preparations. To begin the SEM analysis, ultracentrifuged pellets were fixed with 3.5% glutaraldehyde overnight at 4°C. After removing glutaraldehyde via centrifugation, fixed exosomes were dehydrated with an ascending sequence of ethanol (15%, 30%, 60%, 80%, and 100%). Afterward, the samples were dried at room temperature for 24 hours on an aluminum sheet, and viewed using SEM with gold-palladium sputtering. Moreover, pellet size was measured by Mastersizer 2000E nanoparticle analysis (NPA) with Beckman Coulter (Indianapolis, IN). After exosome precipitation, the pellets were resuspended in 0.5 mL PBS and sent for NPA. Each experiment was carried out in triplicate.

### 2.4 Western blot

Total protein was prepared from exosomes pellet by using a Protein Extraction Kit (Applygen, Beijing, China) and separated via 10% SDS-PAGE, then transferred onto 0.22 μm PVDF membranes. After 3 hours blocking with 5% nonfat milk, membranes were incubated with primary rabbit antihuman antibodies (Abcam, London, UK) for Alix and CD63 (1:2000) overnight. Secondary goat antirabbit HRP-linked antibody (cwbiotech, Beijing, China) was applied for 1 hour (1:10,000) in blocking buffer. Finally, immunoreactive bands were visualized with an ECL kit (Thermo-Fisher, Shanghai, China).

### 2.5 Quantitative RT-PCR (qRT-PCR) preparation

Total RNA was extracted from exosomes pellet with TRIzol reagent (Invitrogen, California). Quantity and quality were assessed through spectrophotometer (260/280 nm) (Thermo-Scientific, Shanghai, China), which selected eligible RNA for deep sequencing analysis or qRT-PCR validation. Subsequently, cDNA was synthesized in the presence of reverse transcription (RT) primer using a SYBR Premix Ex Taq Kit (RiboBio, Guangzhou, China). After the amplification procedure (40 cycles for 10 seconds at 95°C, 30 seconds at 60°C, 1 second at 70°C), miRNA level was quantitatively determined using a real-time RT-PCR kit, in which miR-39-3p was set as an external control in the analysis of each miRNA.

### 2.6 Small RNA deep sequencing

To characterize miRNA expression profiling, small RNA sequencing and data analysis were conducted by a commercial service (RiboBio, Guangzhou, China). Initially, total RNA samples were sent for RT and PCR amplification. After ligation of 5′ and 3′ adaptors to each end, cDNA were separated on a polyacrylamide gel and low-quality reads were excised to produce clean reads (18–30 nucleotide RNA molecules). Clean reads were aligned to a mature miRNA human genome sequence in miRBase (v21) to identify known miRNAs and used for sequence analysis on an Illumina HiSeq 2500 platform. Differential expression of miRNA among 3 groups was analyzed using a cluster analysis and expressed as the heatmaps (red: overexpressed miRNAs; green: underexpressed miRNAs).

### 2.7 Bioinformatic analyses

During Illumina HiSeq 2500 analysis, miRNA expression (reads/million [RPM] clean tags) was normalized with the formula: RPM = (number of reads mapping to miRNA)/(number of reads in clean date) × 10^6^. Next, we calculated the fold changes in expression levels via formula of log_2_^ratios^ (fold change = log_2_^(sample group 1/group 2)^). Significant differences among groups were assessed using edgeR analysis. All the data were expressed as mean ± SD and *P* values below.05 were considered statistically significant.

## 3 Results

### 3.1 Patient collection

**Figure 1:**
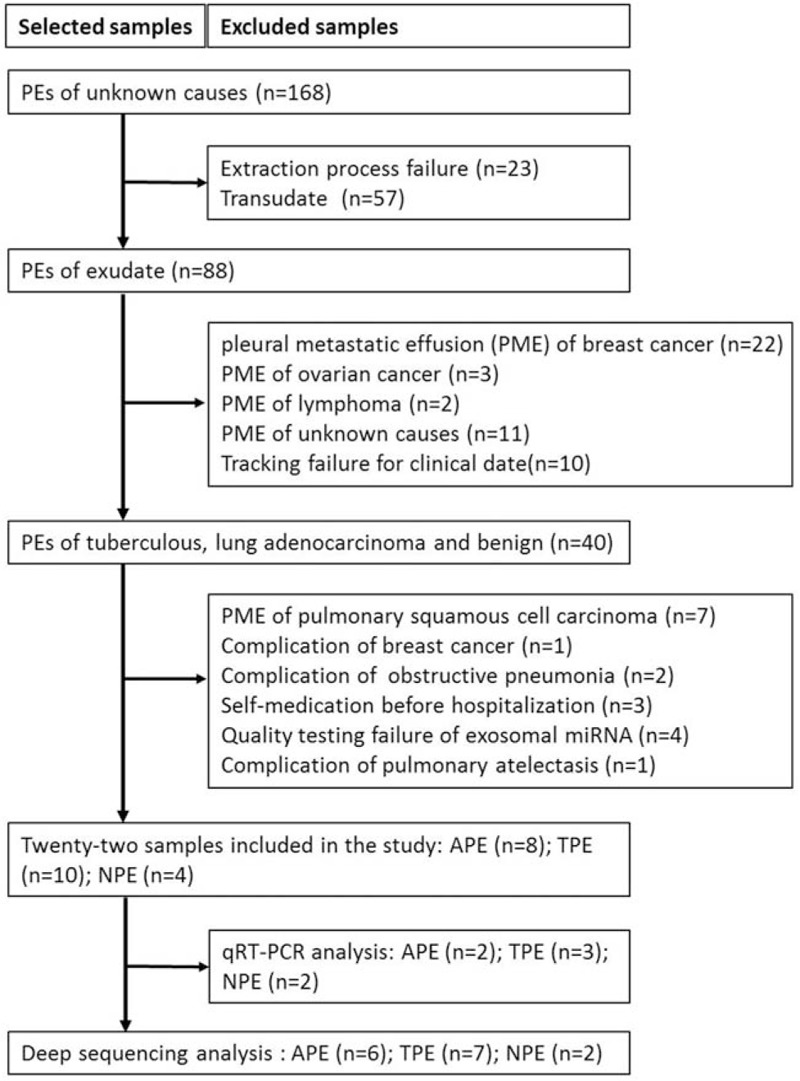
Flow chart of screening eligible samples.

A total of 168 PE samples with unknown causes were successfully collected. We got 22 eligible samples after the exclusion of transudate or PEs with atelectasis, pulmonary embolism, obstructive pneumonia, or other primary tumor disease. Six APE samples were used and mixed to 3 samples in group A for deep sequencing analysis. In the same way, 3 samples from group B mixed from 7 TPE specimens were selected, and 2 NPE samples were kept for deep sequencing analysis. The characteristics of included patients were established in Table 1 and the study design was shown in Fig. 1.

**Table 1:**
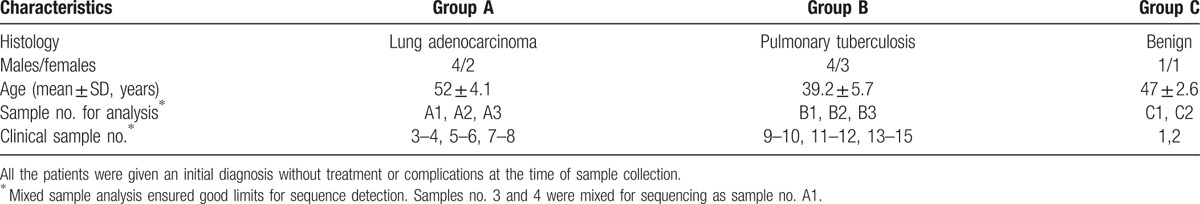
The baseline characteristics of eligible patients in 3 groups.

### 3.2 Characterization of exosomes from PEs

**Figure 2:**
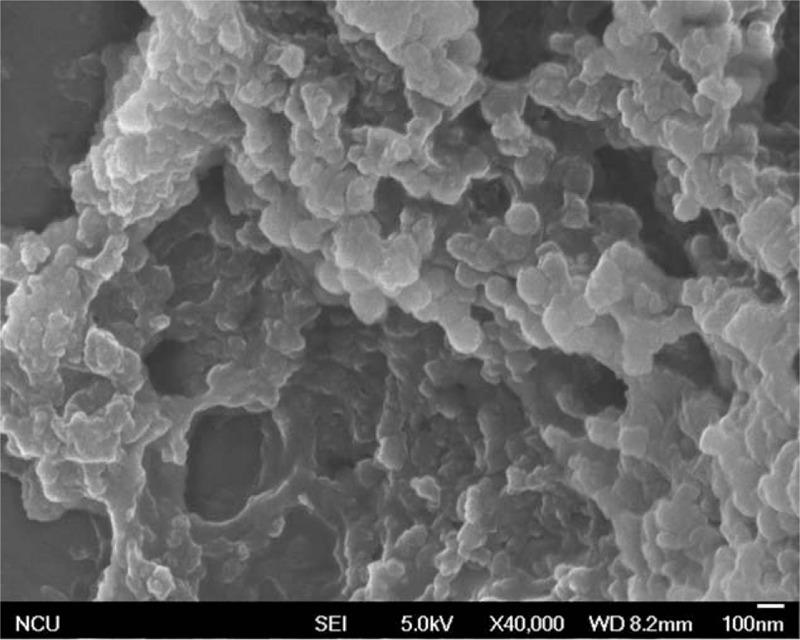
Verification of the exosomal preparation. The morphologic characterization of exosomes purified from PEs was observed by SEM, which showed round structures with heterogeneous size. PE = pleural effusion, SEM = scanning electron microscopy.

**Figure 3:**
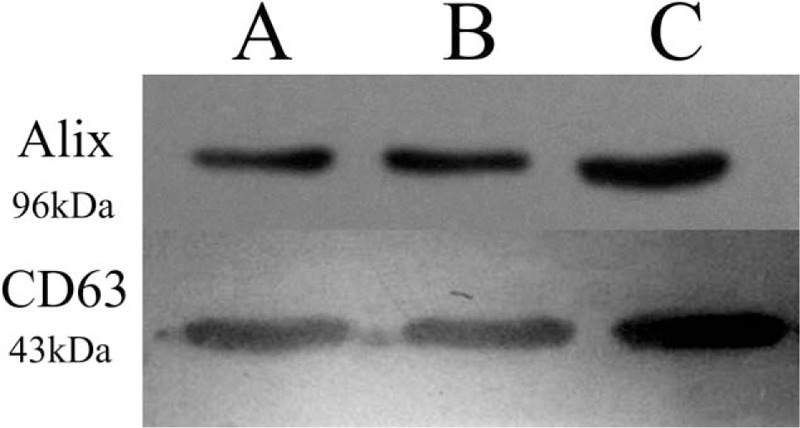
The particle size distribution of exosomes purified from PEs was approximately 30 to 100 nm in diameter. PE = pleural effusion.

**Figure 4:**
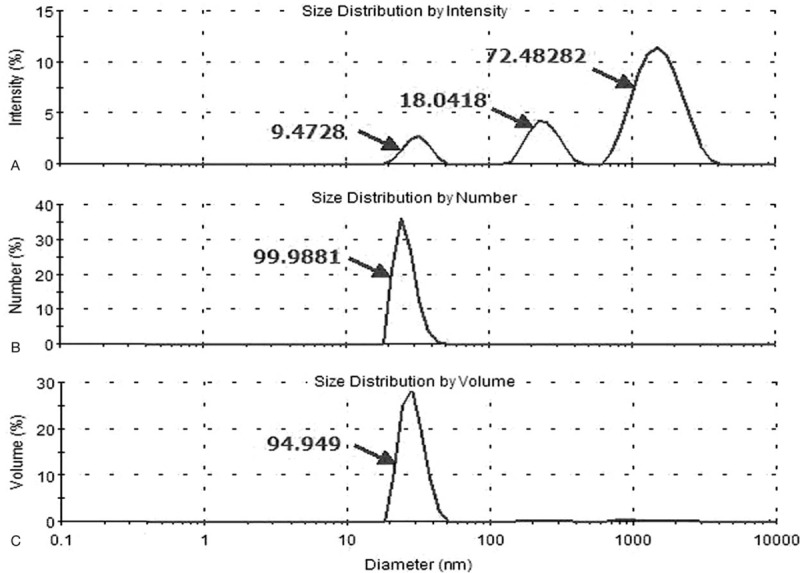
Western blot of 10 to 20 g of proteins extracted from exosomal pellets were positive for exosome-associated proteins (Alix and CD63).

Exosomes were successfully purified through a series of microfiltration and differential centrifugation steps modified by previously description.^[29]^ Results from SEM in Fig. 2 showed round structures with heterogeneous size (30–100 nm), consistent with known vesicular morphology of exosomes.^[33]^ Current criteria to distinguish EVs from exosomes are mainly based on size and density. To do further validation, the particle size distribution of exosomes purified from PEs in NPA results was approximately 30 to 100 nm in diameter (Fig. 3). Pure exosomes were obtained via filtering as described before.^[29]^ Western blot then confirmed the positive typical exosome features of Alix and CD63 (Fig. 4).^[34–36]^

### 3.3 Overview of small RNA sequencing data

Deep sequencing analysis of small RNA libraries for 6 APE, 7 TPE, and 2 NPE samples produced raw, clean, and annotated clean reads, as well as the corresponding total RNA reads in succession (Extended data Table 2). After miRBase (v21) examination corresponding to each sample, more than 470 miRNAs were detected in clinical samples, indicating different miRNA profiles among samples (Extended data Table 3).

### 3.4 MicroRNAs profiling and selection of differential microRNAs

MiRNA-enrichment analysis for exosomes purified from APE, TPE, and NPE was conducted and 171 miRNAs were statistically differentially expressed by applying the screening condition at *P* < .05 (Table 2). Further filtering the miRNAs with the condition of less than 3-fold changes and 100 expression counts yielded 23 highly-represented miRNAs, which could distinguish APE from other groups. Taking the repetition of miRNA and expression balance (std. *P* < .70) of samples in 1 group into consideration, 9 of 23 miRNAs were eventually selected for the identification of APE and all 9 miRNAs were represented in the APE library (Table 3). For example, miR-205-5p owned more than 225 RPM in APE, holding 21-fold changes when compared with corresponding values in NPE libraries, 5.6-fold changes when compared with TPE libraries. The same of great difference, miR-200b-3p, was enriched in exosomes isolated from APE (more than 2612 RPM).

**Table 2:**
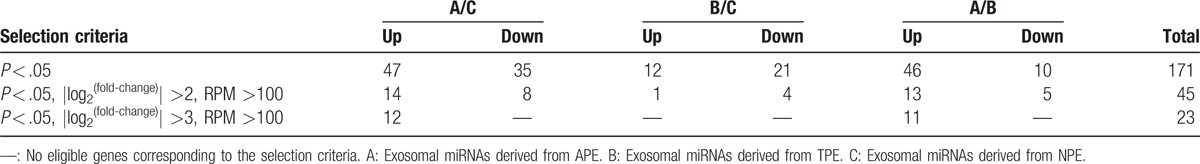
Selection criteria of eligible miRNAs.

**Table 3:**
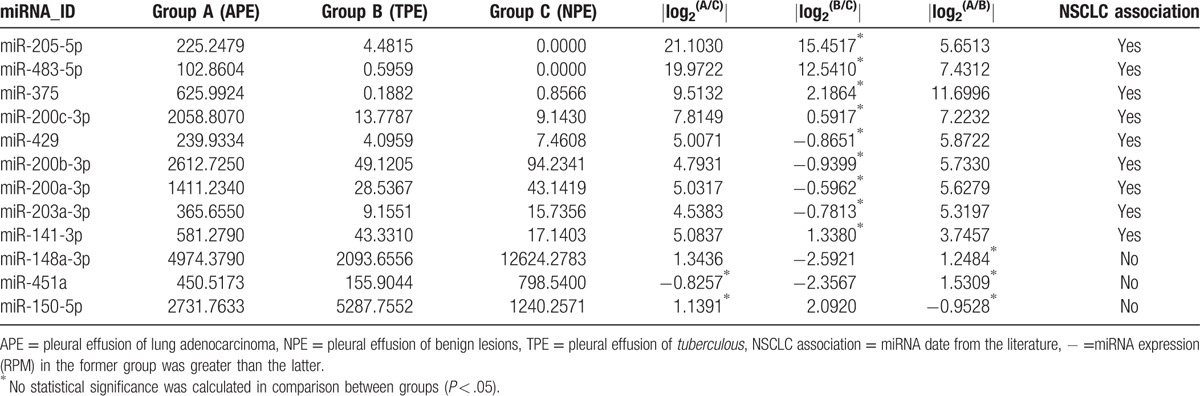
MiRNAs datasets enriched in different exosomes.

**Figure 5:**
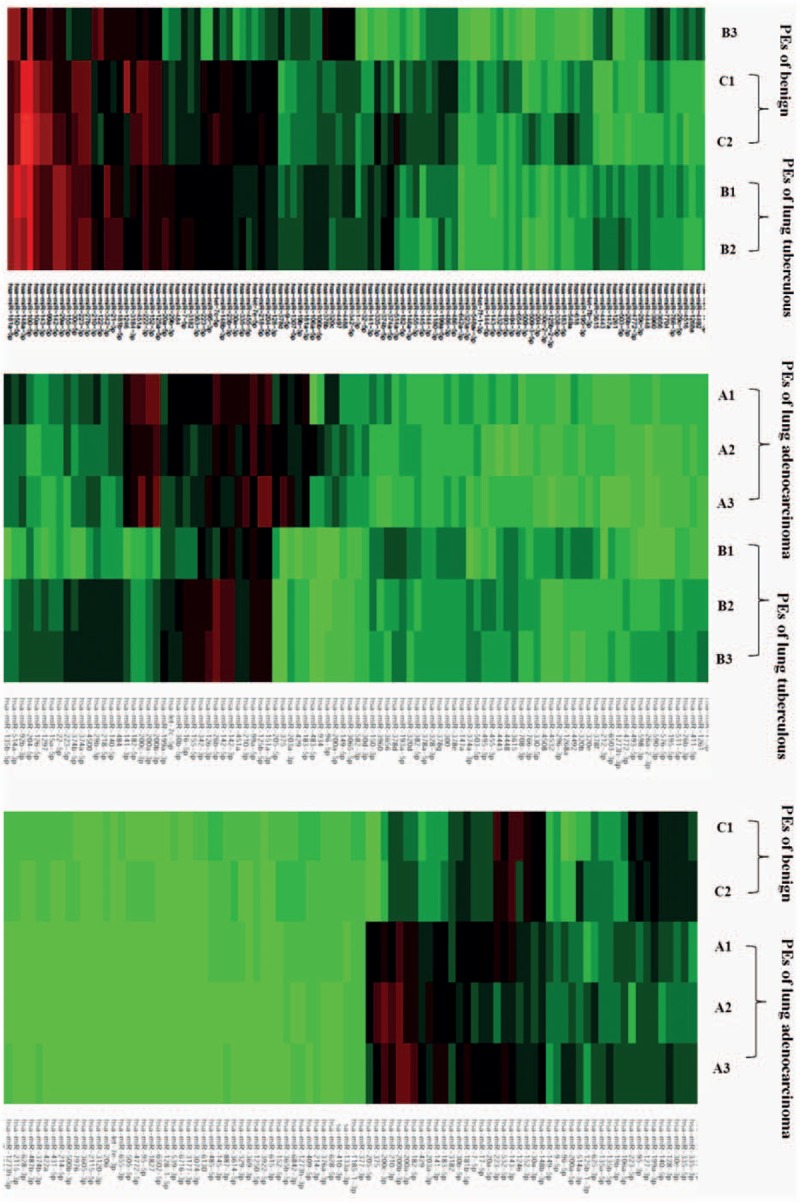
Cluster analysis heatmap of miRNA in 3 kinds of PEs. miRNAs based on global miRNA expression analyses in APE (A1–A3) and NPE (C1–C2). MiRNAs based on global miRNA expression analyses in (A1–A3) APE and (B1–B3) TPE. MiRNAs based on global miRNA expression in (B1–B3) TPE and (C1–C2) NPE samples. Each column represents an miRNA and each row is a sample. Red represents over-expressed miRNAs; green is under-expressed miRNAs. APE = pleural effusion of lung adenocarcinoma, NPE = pleural effusion of benign lesions, TPE = pleural effusion of *tuberculous*,

MiRNAs from TPE and NPE were nearly homogeneous. Three miRNAs (miR-148a-3p, miR-451a, and miR-150-5p) were screened via the second formula in Table 3 and could distinguish TPE from NPE. Cutoff of 2-fold changes was used in this section, which was still sufficient to meet clinically identification criteria. Last, 12 differentiated miRNAs were selected. Cluster analysis, visualized by heatmaps (Fig. 5) showed segregation of the 3 groups. Each column represents a miRNA and each row a sample. Red represents over-expressed miRNAs; green corresponds to under-expressed miRNAs. Significant differences were observed between APE and other groups, whereas TPE and NPE were almost similar except 3 miRNAs (miR-148a-3p, miR-451a, and miR-150-5p).

### 3.5 Quantitative RT-PCR validation

**Figure 6:**
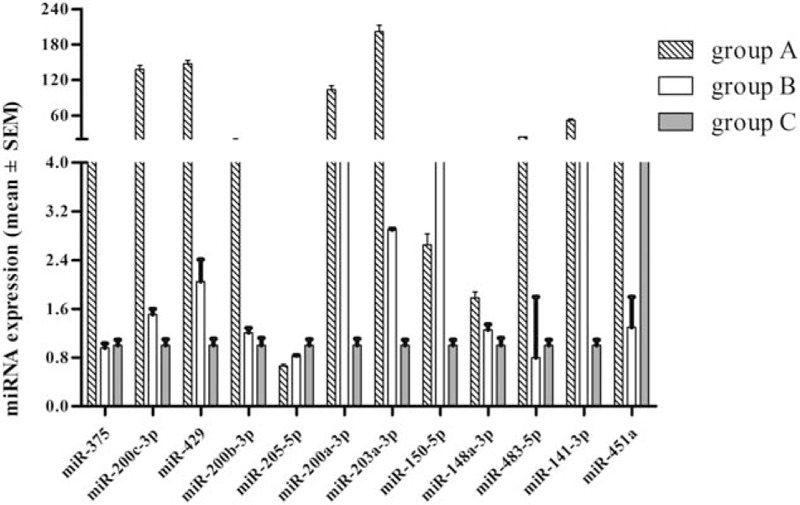
MiRNAs expression overview. MicroRNAs expression from qRT-PCR analysis, with comparative analysis of miRNAs differentially expressed between RT-PCR validation and deep sequencing analysis. RT-PCR = real time polymerase chain reaction, qRT-PCR = quantitative polymerase chain reaction.

Twelve highly expressed miRNAs were selected for qRT-PCR validation (Fig. 6). MiR-39-3p, acted as succeed external control, had the least expression variation in TPE, APE, and NPE: 0.03967, 0.01761, 0.08605 respectively. That is to say, the expression pattern of miRNA among 3 groups can be correctly determined. This figure showed that miR-205-5p was under-expressed in APE but highly expressed in previous deep sequencing analysis of Table 3. MiR-148a-3p was the least expressed according to qRT-PCR but the greatest expressed according to deep sequencing in NPE. This discrepancy may be due to small sample size in the validation phase, which means more samples are needed for validation.

## 4 Discussion

Although it has been 30 years since the discovery of exosomes, more similar observations have reported that exosomes serve important roles in cellular communication and the exosomal process is abnormal in disease.^[37]^ Since its homogeneity over EVs, more and more researchers give priority to exosomes as the study subject. Numerous studies suggest that donor cells can secrete more exosomes compared with healthy cells,^[38–40]^ and the corresponding exosomal content is distinct. According to this feature, many researchers proposed that exosomal miRNAs indicate the disease process,^[41,42]^ can be applied for differentiating diseases,^[43,44]^ which has been equally confirmed in our study. Careful results analysis showed that quite some miRNAs differ among 3 groups of PEs, especially some miRNAs were remarkably highly expressed in 1 group. For this study, 9 miRNAs (miR-200c-3p, miR-200b-3p, miR-200a-3p, miR-429 and miR-141-3p, miR-205-5p, miR-483-5p, miR-375 and miR-203a-3p) were the most abundant profiles in APE when compared with TPE or NPE, and promises to be used to distinguish APE from the other disease states with more validation. Nevertheless, TPE and NPE had nearly similar and low expression of miRNAs compared with APE and only 3 significantly different miRNAs (miR-148a-3p, miR-451a, and miR-150-5p) hold promise to distinguish TPE from NPE.

Five out of the 9 miRNAs significantly differential expressed in APE compared with others (Table 4). Previous studies recommended that miR-200c-3p, miR-200b-3p, miR-200a-3p, miR-429, and miR-141-3p belonged to the miR-200 family and all 5 members were downregulated in cells undergoing epithelial-mesenchymal transition (EMT). Ectopic expression of miR-200 seconds in mesenchymal cells induced mesenchymal-epithelial transition (MET) and miR-200 family members could regulate EMT by repressing expression of ZEB1/ZEB2 (zinc-finger- and homeobox-containing transcriptional regulator delta-crystallin enhancer-binding factor) and Smad-interacting protein 1.^[45–48]^ Namely, high miR-200 expression inhibits lung adenocarcinoma cell invasion and was associated with shorter overall survival in patients with lung adenocarcinoma.^[49,50]^ However, data from systematic analysis of relevance between miR-200 and EMT were variable, which indicated high miR-200 expression in metastatic cancer. Using a mesenchymal-specific Cre-mediated fluorescent marker switch system in spontaneous breast-to-lung metastasis models, Fischer^[51]^ observed that inhibiting EMT by overexpressing miR-200 did not affect the development of lung metastasis. Recent studies established high miR-200 expression in breast or ovarian cancer and ectopic expression of miR-200 conferred metastatic ability in poorly metastatic tumor cells via extracellular vesicles.^[52,53]^ Consistent with these findings, the miR-200 family is abundant in exosomes derived from APE in our study, suggesting a correlation between miR-200 and PE formation.

**Table 4:**
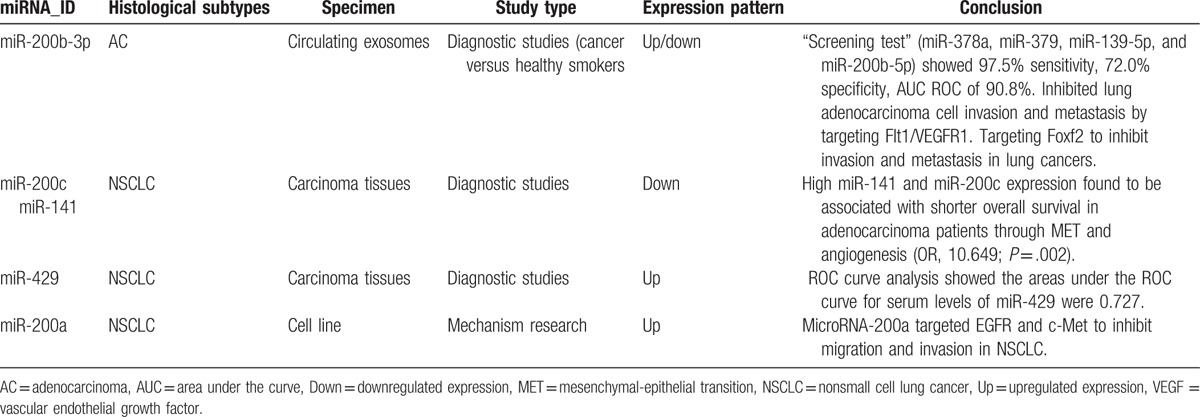
Brief summary of miR-200 family.

The other 4 miRNAs from 9 microRNAs were also previously reported to be related to lung cancer one way or another (Table 5). Data reported here indicated that miR-205-5p was the most expressed and could separate APE from NPE (21-fold change) and separate AE from TPE (5.6-fold change). Yanaihara group come to an agreement with our data, reporting that high miR-205 expression positively correlated with progress of lung cancer and associated with poor survival.^[54]^ Rabinowits’ group reported similarity between circulating exosomal miRNA and tumor-derived miRNA patterns in patients of lung adenocarcinomas,^[42]^ suggesting a diagnostic efficiency of circulating exosomal miR-205 with specific genes isolated from tumor tissues. Similarly, the experimental study of Zhang and even a meta-analysis reported high expression of circulating miR-205-5p in NSCLC.^[26,55]^ Consistent with the miRNAs described above, miR-203a-3p was highly expressed in APE samples and previous studies indicated that miR-203a was less expressed in NSCLC tissues compared with adjacent nonmalignant tissues.^[56]^

**Table 5:**
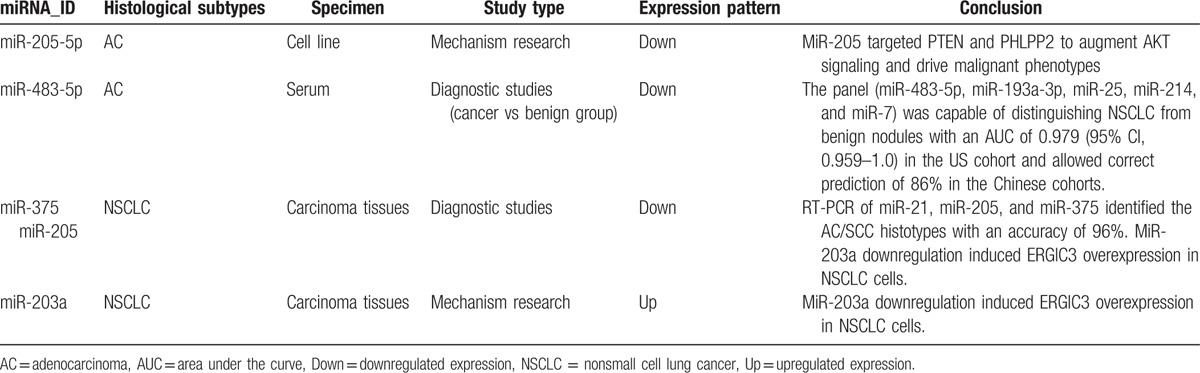
Brief summary of other differential expressed miRNAs.

Most of the above-mentioned miRNAs have been established in our previous study, they owned the same expression pattern with this project. Otherwise, several miRNAs are emerging as different expression level. The differences between 2 studies are assumed to associate with exosomes but EVs, which remains to be proven.

In Wang analysis, miR-483-5p was significantly elevated in NSCLC with more lung adenocarcinoma than squamous cell carcinoma patients, and could be used to distinguish NSCLC from benign nodules (area under the curve 0.979; 95% CI, 0.959–1.0; 86% accuracy).^[57]^ Also previous research suggested miR-483-5p directly targeted 2 putative metastatic suppressors (RhoGDI1 and ALCAM), promoting EMT and increasing invasiveness and metastatic properties of lung adenocarcinoma after activation by the WNT/b-catenin signaling pathway.^[58]^ As far as miR-375, Molina-Pinelo's analysis confirmed that mR-375 was differentially expressed in squamous cell lung cancer compared with adenocarcinoma samples.^[59]^ Claudin-1 is a novel target of miR-375, and high miR-375 expression was correlated with shorter survival time among those with lung adenocarcinoma.^[60–62]^ Moreover, we found miR-9 may be involved in squamous lung cancer by regulating cell cycle-related genes.^[63]^ Benefited from samples limitation of NSCLC to adenocarcinoma, we concluded that miR-483-5p and miR-375 had strong correlation with lung adenocarcinoma, miR-9 has its unique superiority in squamous lung cancer. What is more, miR-148a-3p, miR-451a, and miR-150-5p barely searched in *tuberculous* which prompted new biomarkers in the diagnosis of TPE.

As for the contradictory expression of miR-203a or miR-200 between this study and other literature, we summarized several explanations including inconsistent histological subtypes (NSCLC, adenocarcinoma, or squamous cell carcinoma) across different studies,^[60,61]^ various sample types (serum, tissue, cell lines, EVs, or exosomes); diverse controls (healthy smokers,^[64]^ benign nodules,^[57]^ or para-carcinoma tissues^[56]^), and inconsistent cancer stages (early or advanced during malignant transformation).^[65]^

In conclusion, exosomal miRNAs expression patterns differ among lung adenocarcinoma, tuberculous, and benign samples. Our results show that a group of 9 miRNAs are preferentially sorted into exosomes derived from APE (miR-205-5p, miR-483-5p, miR-375, miR-200c-3p, miR-429, miR-200b-3p, miR-200a-3p, miR-203a-3p, miR-141-3p), and 3 miRNAs (miR-148a-3p, miR-451a, and miR-150-5p) have differential expression between TPE and NPE. Furthermore, miR-483-5p, miR-375, and miR-429 were validated to be associated with lung adenocarcinoma. These miRNAs may hold promise as biomarkers for diagnosing PEs with verification in larger cohort studies.

## Supplementary Material

Supplemental Digital Content
