# Supplementary material for: Identification of differential expressed PE exosomal miRNA in lung adenocarcinoma, tuberculosis, and other benign lesions
Source: Medicine (Baltimore). 2017 Nov 3;96(44):e8361. doi: 10.1097/MD.0000000000008361 (PMC5682784; doi:10.1097/MD.0000000000008361)
Supplement: Supplemental Digital Content [file medi-96-e8361-s001.doc]

Extended data table 1. The difference between exosomes and EVs

|  | **Exosome** | **EVs** |
| --- | --- | --- |
| **Size** | 30-100 nm diameter | Atypical size (30-2500nm) |
| **Origin** | all cell types examined so far | Originally: activated blood platelets and erythrocytes. Recently: mainly tumor cells |
| **Biogenesis** | Endosomal sorting complex required for transport (ESCRT)* | The formation of outward buds |
| **Degradation** | ESCRT-0, -I, -II and –III deliver ubiquitinated proteins to the degradation machinery | Fission and release into the extracellular space |
| **Related proteins** | Susceptibility gene 101 (Tsg101), Alix, ceramide, flotillin, Rab, tetraspannin family, Cholesterol, phosphatidic acid (PA) | Llipid raft domains, RhoA, Calpain, ARF6 |
| **Content** | Protein (surface molecules and exosome markers); Nucleic acids (miRNAs, long non-coding, other non-coding RNAs and mRNA, ssDNA, dsDNA ); Lipids | Tumor-derived molecules, including epidermal growth factor receptor vIII (EGFRvIII), mutant Ras family members, other proteins or transcripts) |
| **Isolation** | 100,000-120,000×g and flow through the 0.22μm filter | 10,000-20,000 × g |

*（Exosomes form within the intraluminal vesicles (ILVs), ESCRT is responsible for accumulation and sorting of molecules channeled into the ILVs）

Extended data table 2 Summary of small RNA sequencing in each sample

|  | A1 | A2 | A3 | B1 | B2 | B3 | C1 | C2 |
| --- | --- | --- | --- | --- | --- | --- | --- | --- |
| Raw reads | 22846576 | 19598859 | 13135527 | 26018757 | 20975855 | 18749728 | 20832102 | 15020010 |
| Clean reads | 20598169 (90.1586%) | 17742284 (90.5271%) | 11817932 (89.9692%) | 23024517 (88.4919%) | 18469538 (88.0514%) | 13983212 (74.5782%) | 19153658 (91.9429%) | 13169996 (87.683%) |
| Annotate clean reads | 20207476 (98.1032%) | 17409567 (98.1247%) | 11576126 (97.9539%) | 22682502 (98.5145%) | 18213792 (98.6153%) | 13552193 (96.9175%) | 18737018 (97.8247%) | 12354376 (93.8069%) |
| Percentage of miRNAs | 7614064 (36.96%) | 5725443 (32.27%) | 2094476 (17.72%) | 7905943 (34.34%) | 6922710 (37.48%) | 2204826 (15.77%) | 6263278 (32.7%) | 3233782 (24.55%) |
| Known miRNA No. | 599 | 524 | 480 | 604 | 579 | 478 | 565 | 493 |

Extended data table 3. Overview of differential microRNAs among three groups

|  | **A VS B** | | **A VS C** | | **B VS C** | |
| --- | --- | --- | --- | --- | --- | --- |
|  | **A** | **B** | **A** | **C** | **B** | **C** |
| Unique miRNA | 2455899 (31.64%) | 3699446 (47.66%) | 2783676 (50.84%) | 1740509 (31.79%) | 3973385 (59.14%) | 1686671 (25.11%) |
| Common miRNA | 950694 (17.36%) | | 1606248 (20.69%) | | 1058370 (15.75%) | |
| Total | 7761593 | | 5474879 | | 6718426 | |
